# Advances in the Treatment of Ischemic Diseases by Mesenchymal Stem Cells

**DOI:** 10.1155/2016/5896061

**Published:** 2016-05-17

**Authors:** Shujing Li, Xianyun Wang, Jing Li, Jun Zhang, Fan Zhang, Jie Hu, Yixin Qi, Baoyong Yan, Quanhai Li

**Affiliations:** ^1^Cell Therapy Laboratory, The First Hospital of Hebei Medical University, Shijiazhuang, Hebei 050000, China; ^2^Department of Radiology, The First Hospital of Hebei Medical University, Shijiazhuang, Hebei 050000, China; ^3^Department of Immunology, Basic Medical College, Hebei Medical University, Shijiazhuang, Hebei 050017, China; ^4^School of Nursing, Hebei Medical University, Shijiazhuang, Hebei 050000, China; ^5^Department of Breast Center, The Fourth Hospital of Hebei Medical University, Shijiazhuang, Hebei 050011, China

## Abstract

Ischemic diseases are a group of diseases, including ischemic cerebrovascular disease, ischemic cardiomyopathy (ICM), and diabetic foot as well as other diseases which are becoming a leading cause of morbidity and mortality in the whole world. Mesenchymal stem cells (MSCs) have been used to treat a variety of ischemic diseases in animal models and clinical trials. Lots of recent publications demonstrated that MSCs therapy was safe and relieved symptoms in patients of ischemic disease. However, many factors could influence therapeutic efficacy including route of delivery, MSCs' survival and residential rate* in vivo*, timing of transplantation, particular microenvironment, and patient's clinical condition. In this review, the current status, therapeutic potential, and the detailed factors of MSCs-based therapeutics for ischemic cerebrovascular disease, ICM, and diabetic foot are presented and discussed. We think that MSCs transplantation would constitute an ideal option for patients with ischemic diseases.

## 1. Introduction

**Figure 1:**
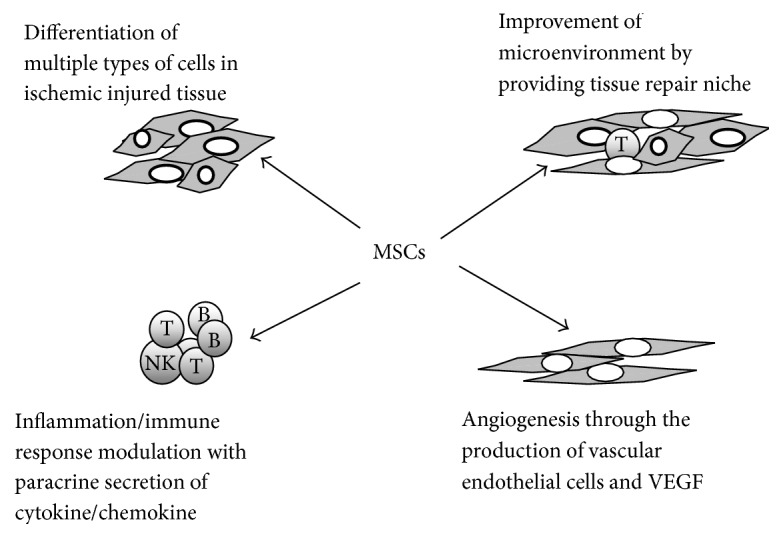


Stem cells (SCs) are defined as undifferentiated long-lived cells with self-renewal ability and multipotency. Depending on the source, SCs can be divided into embryonic, multipotent, and somatic stem cells. Mesenchymal stem cells (MSCs), which are somatic stem cells, were first discovered in the bone marrow by Friedenstein et al. in 1968 [1]. MSCs, with pronounced self-renewal, multilineage differentiation, and immune adjustment abilities, have the advantages of easy separation, high proliferative potentiality, and genetic stability and can proliferate* in vitro*. Cell proliferation pertains to the ability of mesenchymal cell differentiation between multiple passages [2]. Autologous bone marrow MSCs, with the ability of homing to the target, perception of injury signals, migration to the injured site, proliferation, and differentiation, participate in tissue injury repair, achieving effective results [3–6]. MSCs can be obtained from mature bone marrow, fatty tissues, placenta, scalp, pancreatic tissue, endometrium, Wharton's jelly, and umbilical cord blood [7–14]. Under different induction conditions, MSCs can differentiate into bone, cartilage, fat, muscle, tendon, and other mesodermal cells, but also cross mesoderm differentiation, forming various ectodermal cells (e.g., neurons, gliacytes, and skin cells) and endodermal or vascular endothelial cells (e.g., liver and kidney cells) [15, 16]. MSCs secrete multiple biologically active cytokines and growth factors to regulate the local microenvironment and immune response, promoting the repair of damaged tissues. MSCs have been used to treat a variety of ischemic diseases, including ischemic cerebrovascular disease and ischemic cardiomyopathy (ICM), as well as diabetic foot and other diseases. According to the data from animal model and clinical trial of those three ischemic diseases [17–21], MSCs therapy had not induced adverse reactions or made diseases' conditions worsen, which indicated that MSCs therapy is safe. But the efficacy of MSCs therapy was quite different depending on the experimental models. And many factors associated with the efficacy of cell replacement therapy for those ischemic diseases (Table 1), including the route of delivery, MSC type and dose, the timing of transplantation, the particular microenvironment into which cells are delivered, and clinical condition, remain to be addressed. Here we provide the current status and above factors of experimental models and discuss the MSCs therapeutic potential (Figure 1). We believe that MSCs' therapeutics would constitute an ideal option for patients with ischemic diseases.

## 2. Advances in the Treatment of Ischemic Cerebrovascular Diseases by MSC Transplantation

Ischemic cerebrovascular diseases are a group of disorders that lead to cerebral ischemia or necrosis from multiple causes, such as poor blood flow or blood vessel occlusion; they have high incidence and mortality and are the leading cause of long-term disability. However, most of treatments targeted symptoms of cerebral ischemia and effective strategy for its etiology needs to be explored [22]. Over the last decades, MSC therapy has emerged as a particularly appealing option, as it can result in regeneration of neurocyte or rebuilding of nervous tissue after the ischemic insult [23, 24]. Increasing evidence shows that transplanted MSCs significantly promote functional recovery in animal ischemic stroke models [25]. Recently, BM-MSC transplantation was shown to achieve clinical efficacy in patients with ischemic stroke [17, 18].

### 2.1. Transplantation Route

Currently, MSCs transplantation can be performed through three main routes: local lesions, blood circulation (intravenous and intra-arterial injections), and cerebrospinal fluid (intracerebral injection, lumbar injection into the subarachnoid space). All these approaches could deliver MSCs migrating into the injured brain and promote functional improvement in experimental animal models of stroke [26–28]. Intravenous or intra-arterial delivery of MSCs is superior to intracerebral injection: it is less invasive, more extensively neuroprotective, and more easily applied in clinic. Interestingly, Du et al. showed that the intra-arterial (IA) route is a safe and effective way for bone marrow mesenchymal stem cells (BMSCs) transplantation [26]. In the latter study, the therapeutic effects of BMSCs delivered by IA and intravenous (IV) injections in a rat model of transient middle cerebral artery occlusion (MCAO) were compared. Histological analysis demonstrated that the IA route bypasses the pulmonary system, directing the cells to the ischemic parts of the brain more efficiently. Therefore, BMSCs delivered via the IA route were shown to promote angiogenesis and improve functional recovery.

### 2.2. Time and Dose in Cell Transplantation

Wang et al. found that transplantation of BMSCs at 3 and 24 h significantly reduces the lesion volume and improves motor deficits. They also demonstrated that transplanted cells at 1 × 10^6^ to 10^7^ significantly improve functional outcome after stroke [29].

### 2.3. Mechanisms

#### 2.3.1. Cell Homing and Modulation of Inflammatory and Immune Responses

The homing characteristic of MSCs refers to their automatic directional migration to inflammation and injury areas. MSCs have the ability of local chemotaxis to ischemic injury areas, and their mobilization can achieve repair of ischemic injury sites. BMSCs express a variety of cytokines and chemotactic factors (TNF, IL-1B, IL-8, and IL-18) as well as soluble adhesion molecules (L-selectin, E-selectin, P-selectin, and vascular cell adhesion molecules). In a rat model Zhu et al. found that serum IL-1*β*, IL-6, and TNF-*α* levels are rapidly increased, peaking at 2 h after MCAO initiation. Human umbilical cord blood-derived MSC (hUCB-MSC) transplantation markedly and progressively suppresses the ischemia-induced increases of serum IL-1*β*, IL-6, and TNF-*α* levels within 6 h of MCAO reperfusion. Focal cerebral ischemia decreases the serum level of IL-10, which is prevented by hUCB-MSC transplantation. Meanwhile, IL-1*β*, IL-6, IL-10, and TNF-*α* amounts in peri-ischemic brain tissues showed similar changes as in the serum. Moreover, hUCB-MSC transplantation markedly suppresses inflammatory cell infiltration, increases neuronal density, and decreases apoptosis around the ischemic region. The authors concluded that hUCB-MSC transplantation suppresses inflammatory responses and neuronal apoptosis in early stage of focal cerebral ischemia [30]. Jiang et al. transplanted adipose-derived mesenchymal stem cells (ADMSCs) via internal carotid and found that injected cells migrated to the brain infarct region and were mainly localized in the ischemic core and boundary zone of the lesion. These findings suggested that autologous transplantation of ADMSCs attenuates astroglial reactivity, inhibits cellular apoptosis, promotes cellular proliferation, and improves the neurological function after acute ischemic stroke [27]. Chung et al. demonstrated that administration of MSCs after transient GCI provides a dramatic protective effect against hippocampal neuronal death. The latter authors hypothesized that the neuroprotective effects of MSC treatment might be associated with the prevention of blood-brain barrier (BBB) disruption and endothelial damage and decreased neutrophil infiltration [31].

#### 2.3.2. Reduced Cell Apoptosis and Induced Angiogenesis

Gu et al. suggested that neuroprotection by MSCs is attributable to anti-inflammatory and antiapoptotic effects through NF-*κ*B inhibition in a rat model [32]. Tang et al. demonstrated that MSCs protect BBB integrity by reducing astrocyte apoptosis after ischemic attack, due to attenuated inflammatory response and downregulated aquaporin-4 expression via p38 signaling, in a rat model of stroke [33]. Chelluboina et al. demonstrated that intravenous administration of utilized human umbilical cord blood-derived mesenchymal stem cells (hUCB-MSC) after focal cerebral ischemia reduces brain damage by inhibiting apoptosis and downregulating the induced apoptotic pathway molecules [34].

#### 2.3.3. Neurotrophic and Neuroprotective Effects

*(1) Induction of Endogenous Cell Proliferation and Paracrine Factors*. MSCs secrete many kinds of neurotrophic factors, including fibroblast growth factor (FGF), nerve growth factor (NGF), VEGF, and BDNF [35]. Huang et al. suggested that paracrine factors inhibit p38 MAPK and JNK, most likely regulating their downstream targets p53 and STAT1, to promote astrocyte survival associated with GFAP downregulation after ischemic stroke* in vitro* [36]. Bao et al. revealed that BMSC transplantation for the treatment of MCAO rat model could significantly improve neuron function recovery at day 14, compared with control groups treated with normal saline. BDNF, neurotrophin-3, and VEGF expression levels were higher, and proliferation of endogenous cells in the subventricular zone (SVZ) and subgranular zone (SGZ) was also increased in the treatment group, compared with control rats. Moreover, more neural progenitor cells migrated to the ischemic boundary zone (IBZ) and differentiated into matured neuron cells with the result of reduced apoptosis [37]. Liang et al. demonstrated that hypoxic exposure causes VEGF and brain-derived neurotrophic factor upregulation, possibly contributing to neurotrophic and neuroprotective effects in* in vitro* hypoxic cortical neuron culture as well as in rat models of focal cerebral ischemia. The authors demonstrated that L-MSCs can secrete various neurotrophic factors, including vascular endothelial growth factor (VEGF), VEGFR3, brain-derived neurotrophic factor, insulin-like growth factor-2, and hepatocyte growth factor, stimulating neurite outgrowth and protecting neurons against brain ischemic injury through a paracrine mechanism [38].

*(2) Improved Partial Pressure of Oxygen and Better Microenvironment*. Huang et al. showed that M17 neuronal cell proliferation is significantly decreased with increased apoptosis after exposure to oxygen-glucose deprivation (OGD) stress. These effects could be alleviated by coculture with MSCs. TNF-*α* was elevated after OGD stress and returned to normal levels after coculture with MSCs. The authors demonstrated that these effects involve IL-6 and vascular endothelial growth factor signaling pathways, with MSCs having anti-inflammatory properties and the capacity to rescue the injured neurons [39]. Shichinohe et al. found that BMSCs significantly ameliorate glutamate-induced neuronal death and improve the survival of neurons in peri-infarcted areas in a rat model. FISH analysis revealed that approximately half of BMSCs express BDNF and NGF mRNA 2 weeks after transplantation; however, the percentages of BDNF and NGF mRNA-positive cells decreased thereafter. Instead, the percentage of microtubule-associated protein 2-positive BMSCs gradually increased for 4 weeks after transplantation. These authors concluded that BDNF may be a key factor underlying the trophic effects of BMSCs [40].

### 2.4. Clinical Trials and Safety Data

Twenty patients with cerebral arterial thrombosis were randomly divided into two groups by Díez-Tejedor et al. and intravenously injected with allogeneic adipose-derived MSCs or placebo, respectively. Within the first 2 weeks from stroke onset, the dose was 1 million MSCs per kilo of weight, administered at an infusion rate of 4–6 mL/minute. During two years of follow-up, intravenous injection of allogeneic adipose-derived MSCs was found to facilitate neuronal protection and repair, with a satisfactory safety profile [41].

### 2.5. Outcome Difference of MSCs Transplantation in Cell Source

Zacharek et al. suggested that treatment of stroke with MSCs from stroke rats is more effective than with cells from normal animals due to enhanced angiogenesis and arteriogenesis via the Ang1/Tie2 system as well as neurological outcomes [42]. MSCs from stroke rats (1 × 10^6^) or cells from normal animals (1 × 10^6^) were intravenously injected into tail vein at 24 hours after MCAO. A modified Neurological Severity Score evaluation and foot fault tests were performed before MCAO and at 1, 7, and 14 days after MCAO. MSCs from stroke rats significantly promoted functional outcome and enhanced angiogenesis, arterial density, and axonal regeneration compared with those from normal animals. MSCs from stroke rats exhibited increased Angiopoietin-1, Tie2, basic fibroblast growth factor, glial cell-derived neurotrophic factor, vascular endothelial growth factor, and Flk1 gene expression compared with those from normal animals. Using transwell coculture of MSCs with brain-derived endothelial cells, MSCs from stroke rats increased phosphorylated-Tie2 activity in brain-derived endothelial cells and enhanced brain-derived endothelial cells capillary tube formation compared with those from normal animals. Inhibition of Tie2 gene expression in brain-derived endothelial cells using siRNA significantly attenuated MSC-induced capillary tube formation [42]. This data indicated that the outcome of MSCs therapy could depend on the source of cells.

## 3. Advances in the Treatment of Ischemic Cardiomyopathy by MSC Transplantation

Ischemic cardiomyopathy (ICM) is becoming a leading cause of morbidity and mortality worldwide. Common treatment strategies, such as pharmacotherapy, coronary artery bypass grafting (CABG), and coronary artery stent, enable recovery of blood supply to the ischemic regions and relatively alleviate pain and suffering but fail to treat the pathophysiological changes following ischemic injury and regenerate novel muscle tissues. The ideal treatment effect is to induce myocardial regeneration of resident cardiac progenitor cells or other exogenous multipotent stem cells [43]. Stem cell implantation treatment for ICM represents a new hope for patients while facing new challenges. Accumulating evidence suggests that stem cells repair the damaged heart by differentiating cardiac muscle cells, promoting angiogenesis, forcing the proliferation of endogenous cardiac stem cells, and secreting cytokines, chemokines, and growth factors to activate endogenous reparative responses, which inhibit cell apoptosis and fibrosis and improve myocardial contraction [44]. Different cell types, including MSCs, have been used to evaluate the cell-based therapeutic potential.

### 3.1. Transplantation Route

Currently, cardiovascular stem cell transplantation can be performed through four main routes: intramyocardial, intravenous, intracoronary, and percutaneous puncture catheter interventional endocardial injections. A placebo-controlled trial recruited 69 patients with AMI after percutaneous coronary intervention (PCI), in which they were treated by intracoronary delivery of autologous MSCs. MSCs suspension containing 8 × 10^9^-9 × 10^9^ cells was directly injected through an inflated over-the-wire balloon catheter center lumen into the target coronary artery. Three months after transplantation, significant improvements of LVEF and ratio of end-systolic pressure to end-systolic volume were obtained in comparison with the control group, indicating effective abilities of cardiac repair and reverse remodeling after autologous MSCs administration [45]. Hare et al. performed a double-blind, placebo-controlled, dose-ranging (0.5, 1.6, and 5 million cells/kg) safety trial of intravenous allogeneic hMSCs at a rate of 2 mL/min. The primary end point was incidence of treatment-emergent adverse events within 6 months. Intravenous injection of allogeneic MSCs which reduced ventricular tachycardia episodes and increased LVEF within six months produced a similar effect [46]. In addition, a clinical trial assessing intramyocardial injection of autologous MSCs in patients with remote myocardial infarction for 1 year showed a decreased infarct size and improved regional left ventricular (LV) function by testing the peak Eulerian circumferential strain [47]. This suggested that either delivery methods are associated with the therapeutic effects. Taken together, intramyocardial and intracoronary routes are more promising than intravenous injection.

### 3.2. Time and Dose in Cell Transplantation

A clinical trial demonstrated that low-dose MSCs (20 million) result in greater reduction of LV volumes and increased LVEF compared with high-dose groups (100 and 200 million) [48], corroborating another report that low-dose intramyocardial injection of CD34+ cells (1 × 10^5^ cells/kg) causes greater improvement in exercise tolerance and lower weekly angina frequency at both 6 and 12 months compared with the high-dose group (5 × 10^5^ cells/kg) [49]. Random-effects meta-analysis performed on 888 animals in 52 studies suggested that MSC therapy is more effective than BMMNC treatment; sensitivity analysis revealed that efficacy is more pronounced with higher cell numbers (≥10^7^) and late injections (>1 week) [19]. A recent comparative clinical trial found higher efficacy in MSC-treated individuals than the BMMNC-treated group [50]. Infusion time varies from several days to several weeks after acute myocardial infarction (AMI). A study of infusion timing showed no differences in LV function improvement at 4 months after intracoronary infusion of BMMNCs at either 5 to 7 days or 3 to 4 weeks among 200 patients with ST-segment elevation myocardial infarction compared with the control group [51]. The TIME randomized trial showed no significant improvement in global or regional EF in patients with left ventricular dysfunction after intracoronary infusion of 150 × 10^6^ BMMNCs at either 3 or 7 days [52]. However, a pooled analysis of 7 randomized controlled trials assessing 660 patients with AMI indicated that bone MSC infusion at 4 to 7 days after AMI results in greater improvement in LVEF and reduction of LV end-systolic dimensions, compared with infusion within 24 hours, which might be associated with acute inflammatory reactions immediately following AMI [53]. These findings show the importance of the timing-window and dosage of injected cells, which need further exploration in more detailed clinical studies.

### 3.3. Mechanisms

#### 3.3.1. Migration and Differentiation

Studies in animal models of myocardial infarction have demonstrated the ability of transplanted MSCs to engraft and differentiate into myocardial cells, contractile fiber cells, and endothelial cells [54–56]. Quevedo et al. found that transplanted MSCs could differentiate into myocardial cells, contractile fiber cells, and endothelial cells. Twelve weeks after myocardial infarction, female swine received catheter-based transendocardial injections of either placebo (*n* = 4) or male allogeneic MSCs (200 million; *n* = 6); the animals underwent serial cardiac magnetic resonance imaging, and* in vivo* cell fate was determined by colocalization of the Y-chromosome (Y(pos)) cells with markers of cardiac, vascular muscle, and endothelial lineages. MSCs engrafted in infarct and border zones differentiated into cardiomyocytes as ascertained by colocalization with GATA-4, Nkx2.5, and alpha-sarcomeric actin. In addition, Y(pos) MSCs caused vascular smooth muscle and endothelial cell differentiation, contributing to large and small vessel formation. Infarct size was reduced from 19.3 ± 1.7% to 13.9 ± 2.0% (*P* < 0.001), while ejection fraction (EF) increased from 35.0 ± 1.7% to 41.3 ± 2.7% (*P* < 0.05) in MSC group over 12 weeks. Importantly, MSC engraftment correlated with functional recovery in contractility (*R* = 0.85, *P* < 0.05) and MBF (*R* = 0.76, *P* < 0.01) [56].

#### 3.3.2. Secretion of Factors That Promote Angiogenesis, Modulate Inflammation and Immune Response, Increase the Ability of Myocardial Reperfusion, and Reduce Cell Apoptosis

Teng et al. found that the MSC-derived exosomes significantly enhanced the tube formation of human umbilical vein endothelial cells, impaired T-cell function by inhibiting cell proliferation* in vitro*, reduced infarct size, and preserved cardiac systolic and diastolic performance compared with PBS markedly enhancing the density of new functional capillary and hence blood flow recovery in rat myocardial infarction model. They demonstrated that exosomes stimulate neovascularization and restrain the inflammation response, thus improving heart function after ischemic injury [57]. Zubkova et al. found that treatment with TNF-*α* enhances ADSC proliferation and F-actin microfilament assembly and increases cell motility and migration through the extracellular matrix. Exposure of adipose-derived stem cells (ADSCs) to TNF-*α* led to increased mRNA expression of proangiogenic factors (FGF-2, VEGF, IL-8, and MCP-1), inflammatory cytokines (IL-1*β* and IL-6), proteases (MMPs and uPA), and the adhesion molecule ICAM-1. At the protein level, VEGF, IL-8, MCP-1, and ICAM-1 production was also upregulated. Stimulation with TNF-*α* was shown to trigger ROS generation and activate a number of key intracellular signaling mediators known to positively regulate angiogenesis, including Akt, the small GTPase Rac1, ERK1/2, and p38 MAP-kinases. These findings indicated that TNF-*α* plays a role in activating the angiogenic and regenerative potential of ADSCs [58]. Wen et al. indicated that the hypoxia-responsive microRNA-377 directly targets VEGF in MSCs, with knockdown of endogenous microRNA-377 promoting MSC-induced angiogenesis in the infarcted myocardium [59].

Boomsma and Geenen demonstrated that MSCs synthesize and secrete multiple paracrine factors that are able to regulate MSC migration, promote angiogenesis, and reduce apoptosis. While both MCP-1 and PI3-kinase are involved in the protective effect, they act independently. It is likely that multiple prosurvival factors, such as macrophage inflammatory protein-1*α* (MIP-1*α*), MIP-1*β*, and monokine induced by IFN-*γ* (MIG), are secreted by MSCs and act on divergent intracellular signaling pathways [60].

### 3.4. Clinical Trials and Safety

Several clinical trials have assessed the feasibility, safety, and efficacy of MSC transplantation therapy in patients with ICM. Recent studies suggested that patients following AMI show improved LVEF in the bone MSC treatment group versus controls [61, 62]. A recent meta-analysis involving 1255 patients showed moderate quality evidence that bone MSC treatment improves LVEF [63]. Two other meta-analyses evaluated bone MSC therapy for ischemic heart disease and concluded that bone MSC treatment significantly reduces mortality risk, decreases angina episodes per week, and results in an even better quality of life [64, 65]. Therefore, most of the above studies showed to some extent improvements of cardiac function as well as alleviation of left ventricular dilation and remodeling in ICM patients. However, a study published in JAMA in 2014 revealed that although transendocardial stem cell injection with MSCs improves 6-minute walk distance, regional myocardial function, and peak Eulerian circumferential strain at the site of injection and reduces infarct size as a percentage of LV mass, no changes were observed in left ventricular chamber volume and ejection fraction [50]. All the above studies demonstrated the safety of MSC grafts, and no severe adverse events were observed in patients with ICM diseases.

## 4. Advances in the Treatment of Diabetic Foot Ulceration by MSC Transplantation

Diabetes is reaching epidemic proportions worldwide [81]. Diabetic foot syndrome (DFS), which includes ulcerations, infections, and Charcot osteoneuropathy, is the most frequent reason for hospitalization, and nonhealing ulcerations may lead to amputation in spite of current standards of care. MSCs provide a novel therapeutic option and have been shown to be beneficial in diabetic wound healing.

### 4.1. Transplantation Route

Adequate homing of MSCs to the injured tissue is important for effective therapy. In most studies, MSCs were administered through a standard intravenous route. A disadvantage of the systemic intravenous delivery of MSCs can be low uptake at the site of injury. Indeed, MSCs were found at low or very low frequencies in target organs [82–84]. Zonta et al. showed that intra-arterial administration of MSCs is the most effective route to achieve immunomodulatory effects in experimental kidney transplantation [76]. Ho et al. demonstrated that multiple intravenous transplantations of MSCs effectively restore long-term blood glucose homeostasis for 15 weeks in STZ-induced diabetic mice; thus, multiple intravenous MSC transplantations may serve as a new therapeutic strategy for DM patients [75]. Lee et al. indicated that multiple intramuscular ATMSC injections constitute a safe alternative to achieve therapeutic angiogenesis in CLI patients, who are refractory to other treatment modalities [70].

### 4.2. Mechanisms

MSCs were shown to induce angiogenesis in ischemic limb tissues, increasing blood flow, granulation tissue formation, epidermal cell regeneration, and wound healing, which improve limb salvage rate [66, 78]. The normal wound healing is a dynamic and complicated process, encompassing vascular closure, solidification of blood clots, acute inflammatory reactions, cell migration, cell proliferation, cell differentiation, angiogenesis, epithelium formation, and extracellular matrix synthesis and reconstruction. However, diabetes is the prototype model of impaired wound healing. Predisposition to ulceration in individuals with diabetes has multifactorial and interrelated causes, including persistent infection, tissue ischemia, necrosis, exudation, and overexpression of inflammatory factors [79]. MSCs provide a novel therapeutic option and are beneficial in diabetic wound healing. In the field of wound healing, use of MSCs is effective through modulating inflammation, extracellular matrix production, migration of keratinocytes, and angiogenesis for cell therapies. The mechanisms underlying the beneficial effects in wound healing include paracrine secretion of growth factors and chemokines requisite for wound healing and MSC differentiation into keratinocytes and endothelial cells required for wound healing and angiogenesis. It has been demonstrated that the healing difficulty of chronic diabetic wounds is mainly ascribed to the lack of angiogenesis [80]. Nie et al. [77] found that adipose-derived stem cells (ASCs) express VEGF-A, HGF, and fibroblast growth factor-2. A total of 1 × 10^6^ ASCs were injected intradermally around the wound at eight injection sites in diabetic rats model. The wounds healed faster and the histological observation showed that tissue regeneration was much greater. Immunofluorescent analysis indicated that GFP-expressing ASCs were costained with pan-cytokeratin and CD31, respectively, indicating spontaneous site-specific differentiation into epithelial and endothelial lineages. ASCs were found to secrete angiogenic cytokines* in vitro* and* in vivo*, including VEGF, HGF, and FGF2, which increase neovascularization and enhance wound healing in injured tissues. These results demonstrated that ASCs significantly enhanced neovasculogenesis and reduced the time to wound closure. Wang et al. [72] revealed that HUMSC implantation plays a positive role in promoting ulceration recovery in rats with diabetic foot. RT-PCR and Western blot showed that VEGF was significantly upregulated at the gene and protein levels (*P* < 0.05). VEGF immunostaining was positive in blood vessels, whose densities in the HUMSC group were increased significantly (*P* < 0.05). You et al. carried out an* ex vivo* study of human umbilical cord derived MSCs for trauma repair and showed that hUCB-MSCs may have greater capacity for diabetic wound healing than allogeneic or autologous fibroblasts, especially in improving angiogenesis [73]. MSCs could partially repair the function of neurons. Xia et al. [74] indicated that hMSCs-UC treatment partially reverses neuronal degeneration and femoral nerve function in rats with diabetic foot ulceration, which might be due to upregulated nerve growth factor and dramatic angiogenesis in FN-innervated gastrocnemius, consequently reversing neuronal structure and function, preventing or curing foot ulceration. MSCs could facilitate wound healing in diabetic mice by improving keratinocyte cell function. Kato et al. [67] suggested that the impaired healing process in diabetic rats can be ameliorated by transplantation of BM-MSCs. This amelioration might result from modified keratinocyte function. In keratinocytes cultured with MSC-CM, the decreased pFAK levels in high glucose conditions were restored, and MMP2, EGF, and IGF-1 levels increased. Shen et al. [68] suggested that NT-3 significantly promotes hMSC secretion of VEGF, NGF, and other vasoactive factors, accelerating wound healing by inducing angiogenesis through improved activation of vascular endothelial cells. The hMSCs stimulated by NT-3 can produce materials that accelerate wound healing in the diabetic foot and other ischemic ulcers.

### 4.3. Methods to Improve the Therapeutic Efficacy of MSCs

The function of MSCs is impaired under high glucose conditions [71, 85, 86]. It was previously shown that several cell types infused into the body rapidly undergo cell death [87]. Kim et al. found that MSCs might necrotize or undergo apoptosis within 24 hours of transplantation due to ischemia, hypoxia, and lack of nutrients in the harsh environment [88]. In addition, it has been reported that low oxygen pretreatment of bone marrow MSCs can improve survival rate after transplantation [69, 89, 90].

Biomaterials such as collagen allow targeted delivery and positioning of high numbers of cells at the wound site. Therefore, use of biomaterials in conjunction with stem cell therapy* in vivo* may ensure sustained cell viability and functionality. Allogeneic nondiabetic bone marrow derived MSCs were seeded in a collagen scaffold, and topical application of 1,000,000 MSCs on the collagen scaffold resulted in increased wound closure and enhanced angiogenesis [91]. Autologous MSCs derived from the patient's bone marrow were seeded directly to the wound and injected into wound edges and finally covered with a prepared autologous biograft. The wound showed a steady overall decrease in size and an increase in dermal vascularity and thickness after 29 days of combined treatment. The therapeutic effects of collagen and other biomaterial seeded MSC therapy in diabetic wound healing are currently being investigated [20].

### 4.4. Clinical Trials and Safety

Forty-one diabetic foot patients were randomly divided into two groups and underwent intramuscular injection of bone marrow mesenchymal stem cells (BMMSCs) and bone marrow-derived mononuclear cells (BMMNCs); ulcer healing rate in the BMMSC group was significantly higher than that obtained with BMMNCs at 6 weeks after injection (*P* = 0.022), reaching 100% 4 weeks earlier than the BMMNC group [21]. After 24 weeks of follow-up, improvements in limb perfusion induced by BMMSC transplantation were more significant than those obtained with BMMNCs in terms of painless walking time (*P* = 0.040), ankle-brachial index (ABI) (*P* = 0.017), transcutaneous oxygen pressure (TcO(2)) (*P* = 0.001), and magnetic resonance angiography (MRA) analysis (*P* = 0.018). The authors concluded that BMMSC therapy may be better tolerated and more effective than BMMNC treatment for increasing lower limb perfusion and promoting foot ulcer healing in diabetic patients with CLI.

## 5. Perspectives

Many parameters need to be further investigated to achieve the maximum effects regarding the optimal separation process, ideal cell type, time-window for transplantation, appropriate cell dosage, optimal route of delivery, and clinical indications. We believe that through continued and collaborative efforts MSCs transplantation will yield a satisfactory response in ischemic disease patients.

## Figures and Tables

**Table 1:** Summary of important factors and efficacy of MSCs therapeutics for ischemic cerebrovascular disease, ischemic cardiomyopathy ICM, and diabetic foot from recent publications.

Name of ischemic disease	MSC source	Transplantation route	Transplantation timing and dose	Major efficacy	Mechanisms
Ischemic cerebrovascular disease	Bone marrow MSCs [26, 29, 37, 42], adipose-derived MSCs [27, 31, 41], and umbilical cord MSCs [30]	Intracranial transplantation [28], intracerebral injection [26], and intracerebral injection [27]	Within 24 h after stroke, 10^6^~10^7^ MSCs [29]	Angiogenesis induction, neuron protection, and inflammation suppression [26, 27, 30, 35–40]	Growth factors secretion, endogenous cell proliferation, and inflammatory cytokine regulation [27, 30, 36–40]

Ischemic cardiomyopathy	Bone marrow MSCs [45, 54, 55, 64, 65],	Intracoronary delivery [45], intravenous injection [46], and intramyocardial injection [47]	Within one week after AMI [51–53], 10^7^~10^8^ MSCs [48, 49, 52]	Improvement of 6-minute walk distance, regional LV and regional myocardial function, infarct size reduction, and angiogenesis induction [44–47, 50, 58, 59]	Cardiac tissue cells differentiation and proliferation, secreting cytokine/chemokine/ growth factor secretion, and inflammation/immune response modulation [44, 54–60]

Diabetic foot	Bone marrow MSCs [66–69], adipose-derived MSCs [70, 71], and umbilical cord MSCs [72–74]	Multiple intravenous delivery [75], intra-arterial transplantation [76], and multiple intramuscular injection [70]	After ischemic limb, 10^6^~10^7^ stem cells [77]	Angiogenesis, neovasculogenesis granulation tissue formation induction, epidermal cell regeneration, and wound healing improvement [66, 67, 70, 72–74, 77–80]	Inflammation regulation, extracellular matrix production, paracrine secretion of growth factor/chemokine, and keratinocytes and endothelial cells differentiation [67, 68, 72–74, 77, 79, 80]

MSCs: mesenchymal stem cells, AMI: acute myocardial infarction, and LV: left ventricular.
